# Opioid Prescribing for Osteoarthritis: Cross-Sectional Survey among Primary Care Physicians, Rheumatologists, and Orthopaedic Surgeons

**DOI:** 10.3390/jcm12020589

**Published:** 2023-01-11

**Authors:** Thomas J. Schnitzer, Rebecca L. Robinson, Lars Viktrup, Joseph C. Cappelleri, Andrew G. Bushmakin, Leslie Tive, Mia Berry, Chloe Walker, James Jackson

**Affiliations:** 1 Feinberg School of Medicine, Northwestern University, Chicago, IL 60611, USA; 2 Value, Evidence and Outcomes, Eli Lilly and Company, Indianapolis, IN 46285, USA; 3 Neuroscience, Eli Lilly and Company, Indianapolis, IN 46285, USA; 4 Statistical Research and Data Science Center, Pfizer Inc., New York, NY 10017, USA; 5 Internal Medicine, Global Medical Affairs, Pfizer Inc., New York, NY 10017, USA; 6 Real World Research, Adelphi Real World, Bollington SK10 5JB, UK

**Keywords:** addiction, centers for disease control and prevention, prescription analgesic, real-world clinical practice, tramadol, treatment guidelines, treatment patterns

## Abstract

Opioids are often prescribed for osteoarthritis (OA) pain, despite recommendations to limit use due to minimal benefits and associated harms. This study aimed to assess physicians’ practice patterns and perceptions regarding opioids by specialty one year following the Centers for Disease Control and Prevention (CDC) published guidance on opioid prescribing. The 139/153 (90.8%) physicians who reported prescribing opioids in the previous year reported decreased prescribing for mild OA (51.3%, 26.5% and 33.3% of primary care physicians, rheumatologists, and orthopaedic surgeons, respectively), moderate OA (50.0%, 47.1% and 48.1%) and severe OA (43.6%, 41.2% and 44.4%). Prescribing changes were attributed to the CDC guidelines for 58.9% of primary care physicians, 59.1% of rheumatologists, and 73.3% of orthopaedic surgeons. Strong opioids were mostly reserved as third-line treatment. Although treatment effectiveness post-CDC guidelines was not assessed, perceptions of efficacy and quality of life with opioids significantly differed across specialties, whereas perceptions of safety, convenience/acceptability and costs did not. Physicians generally agreed on the barriers to opioid prescribing, with fear of addiction and drug abuse being the most important. Across specialties, physicians reported decreased opioid prescribing for OA, irrespective of OA severity, and in most cases attributed changes in prescribing to the CDC guideline.

## 1. Introduction

Opioids are effective for the management of acute musculoskeletal pain [1,2], although there is little evidence specific to their use for osteoarthritis (OA) pain. Opioids have minimal efficacy for chronic OA pain, especially in the longer term, and are associated with considerable safety and tolerability issues [3,4,5,6,7]. Consequently, treatment guidelines for OA specify opioids should only be used after other options have been exhausted [8,9,10,11], and only at the lowest dose and for the shortest duration possible [11]. Despite this, both tramadol and nontramadol opioids are often prescribed for OA pain [12,13,14].

Opioid use is associated with considerable risks of addiction, misuse, and mortality [15,16,17]. There are few robust opioid abuse/addiction data specific to patients with OA [4], but a study based on Optum claims data of patients with OA of the hip or knee found opioid use disorder in 1.2% and 4.2% of patients initially treated with tramadol or nontramadol opioids, respectively [12]. Hospitalizations for opioid use disorder in patients with OA increased 14-fold in the United States (US) from 1998–2000 to 2015–2016, based on US National Inpatient Sample data [18].

In March 2016, the Centers for Disease Control and Prevention (CDC) published guidance on prescribing opioids for chronic pain outside of active cancer treatment, palliative care, and end-of-life care [19,20]. This guideline was intended principally for primary care physicians to assist them in communicating the risks and benefits of opioids to patients, improving treatment outcomes, and reducing opioid-related harms. Opioid prescribing rates for OA were stable in the US during 2007–2014 [13,14]. Since the publication of the CDC 2016 guideline, physicians’ treatment patterns regarding the use of opioids for patients with OA have not been widely reported.

The objective of this study was to assess US physicians’ practice patterns and perceptions regarding opioid prescribing for OA, across three specialties (primary care physicians, rheumatologists, and orthopaedic surgeons), one year after the publication of the CDC 2016 guideline.

## 2. Materials and Methods

Data were collected from February to May 2017 using the Adelphi Disease Specific Programme (DSP) [21] for OA, a point-in-time (cross-sectional) survey of physicians in the US. Participating physicians, identified from public lists of healthcare providers, were screened by telephone. Primary care physicians, rheumatologists and orthopaedic surgeons treating at least 10 patients with OA in a typical month were eligible. A predetermined quota per specialty ensured the study population reflected real-world clinical practice in the US.

### 2.1. Outcomes

Data collected included physician demographics, practice characteristics, and responses to practice-level questions relating to their prescribing of opioids and other medications for OA.

#### 2.1.1. Prescribing Changes

Physicians reported any changes in prescribing over the previous year, in response to, “How has your prescribing of opioid drugs changed in the last year for mild, moderate and severe osteoarthritis patients?” (Options for each patient severity category: “decreased greatly”, “decreased somewhat”, “remained the same”, “increased somewhat”, or “increased greatly”; or “I have never prescribed opioids for any of my osteoarthritis patients in the past year”). Those that had increased or decreased their prescribing were asked, “Is your change in opioid prescribing related to the 2016 update to the CDC Guideline for Prescribing Opioids for Chronic pain?” (Options: “yes”, “no” or “I am unaware of these guideline changes”).

#### 2.1.2. OA Severity

Physicians were asked, “What proportion of your mild, moderate and severe osteoarthritis patients receive an opioid-based treatment?” and responded by providing a proportion for each patient severity category for each of weak opioids and strong opioids. The questionnaire did not specify definitions for the severity of OA (mild vs. moderate vs. severe) or type of opioid (weak vs. strong). Physicians were asked, “Do you ever adopt an opioid dose-sparing approach with your patients?” (Options: “yes”, “no” or “I have never heard of this approach”). 

#### 2.1.3. Treatment Strategies

Typical strategies for treating OA were determined, with physicians being asked, “On the grid below are a list of treatment options recommended and/or prescribed by physicians to help control the symptoms of a patient’s osteoarthritis condition. Thinking about a patient who was diagnosed with [mild or moderate/severe] osteoarthritis, what would your typical treatment strategy be at each therapy stage, as treatment options fail?” For each patient category (mild OA and moderate/severe OA), for each of first, second, third and fourth-plus treatment lines, the treatment choices were: lifestyle changes, e.g., diet/exercise; over-the-counter therapy; dietary supplements; nonselective nonsteroidal anti-inflammatory drug (NSAID); selective NSAID; other nonopioid analgesics; weak opioid analgesic; strong-acting opioid analgesic; corticosteroid; topical analgesic; surgery; none; and other. Treatment choices could be selected for more than one treatment line. 

#### 2.1.4. Barriers to Opioid Prescribing

Barriers to opioid prescribing were investigated with the question, “Thinking about possible barriers that might exist when you are considering prescribing an opioid drug, please allocate 100 points across the areas on the grid below in relation to how important you consider each to be as a potential barrier to prescribing opioids. If you think one area is the only barrier, give it 100 points, if you think several areas are barriers distribute the 100 points accordingly” (options: fear of addiction, side effects, tolerance, lack of efficacy as a chronic osteoarthritis pain treatment, patient preference [i.e., patient refusal of opioid], a general lack of comfort in prescribing opioids, fear of drug abuse, fear of drug diversion, guidelines/local restrictions, no barriers at all, or other). Physicians also recorded their level of agreement with the statement, “I have concerns of patient dependence with drugs” (options: strongly disagree, tend to disagree, tend to agree, strongly agree).

#### 2.1.5. Attributes of OA Medications

Physicians rated the performance of treatments for OA-related pain (nonselective NSAID, selective NSAID, nonopioid analgesic, weak opioid, strong opioid, injectable corticosteroid, topical analgesic) against 31 attributes encompassing efficacy, safety, convenience/acceptability, cost and quality of life using a 7-point scale transformed to scores of −3 (performs very badly) to +3 (performs very well).

### 2.2. Statistical Analyses

Responses across physician specialties were analysed using one-way analysis of variance (continuous variables) or chi-square test (categorical variables). In the contingency table analysis with an expected cell count of less than five, Fisher’s exact test (for 2-by-2 tables) [22] or Fisher’s generalised exact test was used (for r-by-c tables, where r or c or both exceed 2) [23]. All data were managed and analysed using SPSS version 7.5 (SPSS Inc., Chicago, IL, USA) and Stata version 17.0 (StataCorp, College Station, TX, USA). *p* < 0.05 was considered statistically significant.

## 3. Results

Of the 153 participating physicians, 81 (52.9%) were primary care physicians, 35 (22.9%) were rheumatologists and 37 (24.2%) were orthopaedic surgeons. Most were male (72.5%), in private practice (84.8%) and had been in practice for ≥15 years (77.8%) (Table 1). When asked about guidelines for treating patients with OA, 45.8% of physicians reported they always follow guidelines (Table 1). The proportion of the patients they considered to have severe OA was 21.1% for primary care physicians, 28.0% for rheumatologists and 25.5% for orthopaedic surgeons (Table 1). 

### 3.1. Prescribing Changes

Overall, 14 physicians reported that they had not prescribed opioids (3.7% of primary care physicians, 2.9% of rheumatologists and 27.0% of orthopaedic surgeons) and 139 that they had prescribed opioids in the previous year, with a significant difference across specialties (Table 2). A total of 93 physicians reported their opioid prescribing had changed (either decreased or increased).

**Figure 1:**
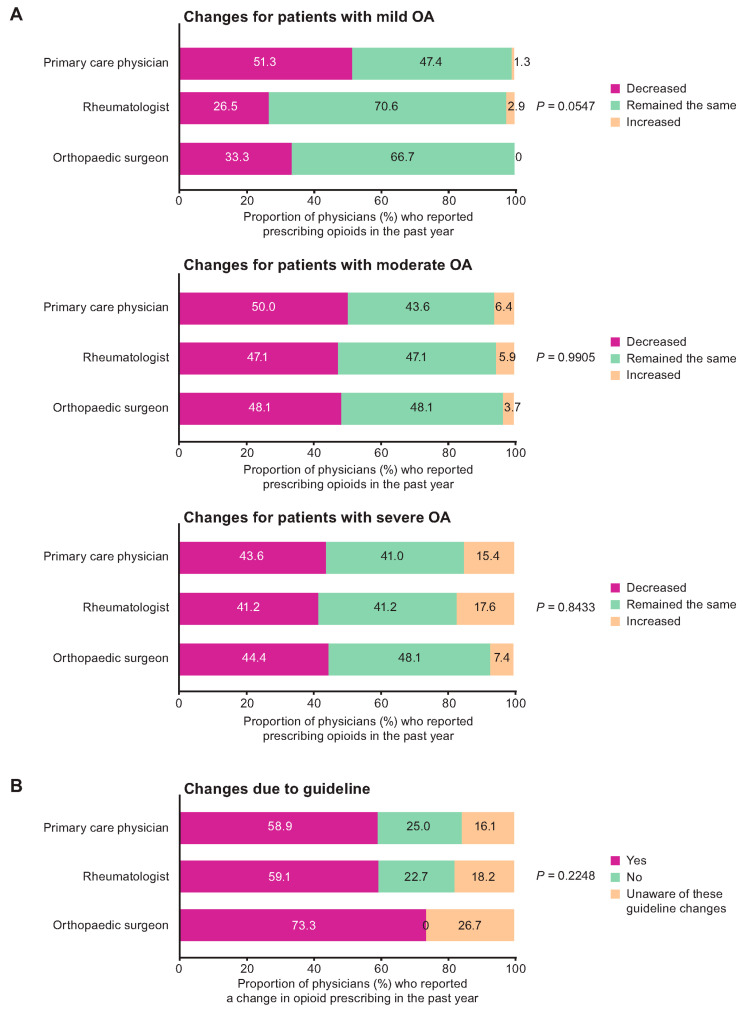
(**A**) Changes in prescribing of opioids for mild, moderate, and severe OA in the past year and (**B**) changes due to CDC 2016 guideline. *p* value for comparison across physician specialties. Figure 1A includes only physicians who prescribed opioids in the past year: n = 78 (primary care), n = 34 (rheumatologist), n = 27 (orthopaedic surgeon). Figure 1B includes only physicians whose prescribing of opioids changed over the past year: n = 56 (primary care), n = 22 (rheumatologist), n = 15 (orthopaedic surgeon). CDC, Centers for Disease Control and Prevention; OA, osteoarthritis.

Across all three specialties, among those physicians who reported prescribing opioids in the past year, decreased opioid prescribing was reported by 41.7% (for mild OA), 48.9% (for moderate OA) and 43.2% (for severe OA); opioid prescribing was reported to have remained the same by 56.8% (for mild OA), 45.3% (for moderate OA) and 42.4% (for severe OA); and increased opioid prescribing was reported by 1.4% (for mild OA), 5.8% (for moderate OA) and 14.4% (for severe OA). All three specialties who reported prescribing opioids in the past year reported reduced prescribing for mild OA (51.3%, 26.5% and 33.3% of primary care physicians, rheumatologists, and orthopaedic surgeons, respectively), moderate OA (50.0%, 47.1% and 48.1%, respectively) and severe OA (43.6%, 41.2% and 44.4%, respectively) (Figure 1A). Reported changes in opioid prescribing did not differ significantly across specialties (Figure 1A). 

Among physicians who reported a change in opioid prescribing, the majority (58.9% of primary care physicians, 59.1% of rheumatologists and 73.3% of orthopaedic surgeons) attributed the change to the CDC 2016 guideline (Figure 1B). Across specialties, 18.3% of physicians (including 16.1% of primary care physicians, 18.2% of rheumatologists and 26.7% of orthopaedic surgeons) who reported a change in opioid prescribing were unaware of the guideline (Figure 1B). There were no significant differences across specialties with respect to the impact of the CDC 2016 guideline (Figure 1B). 

### 3.2. OA Severity

Overall, physicians reported that few patients with mild or moderate OA were prescribed weak (5.3% or 15.6%) or strong (2.3% or 9.9%) opioidsrespectively, with no significant differences in prescribing across specialties (Table 2). Physicians reported that among patients with severe OA, 25.3% and 23.7% were prescribed weak and strong opioids, respectively (Table 2). Significant differences across specialties were found in the proportion of patients with severe OA reported to be prescribed weak opioids (28.2%, 28.4% and 15.9% for primary care physicians, rheumatologists, and orthopaedic surgeons, respectively [*p* = 0.0030]), but not strong opioids (Table 2). 

### 3.3. Treatment Strategies

**Figure 2:**
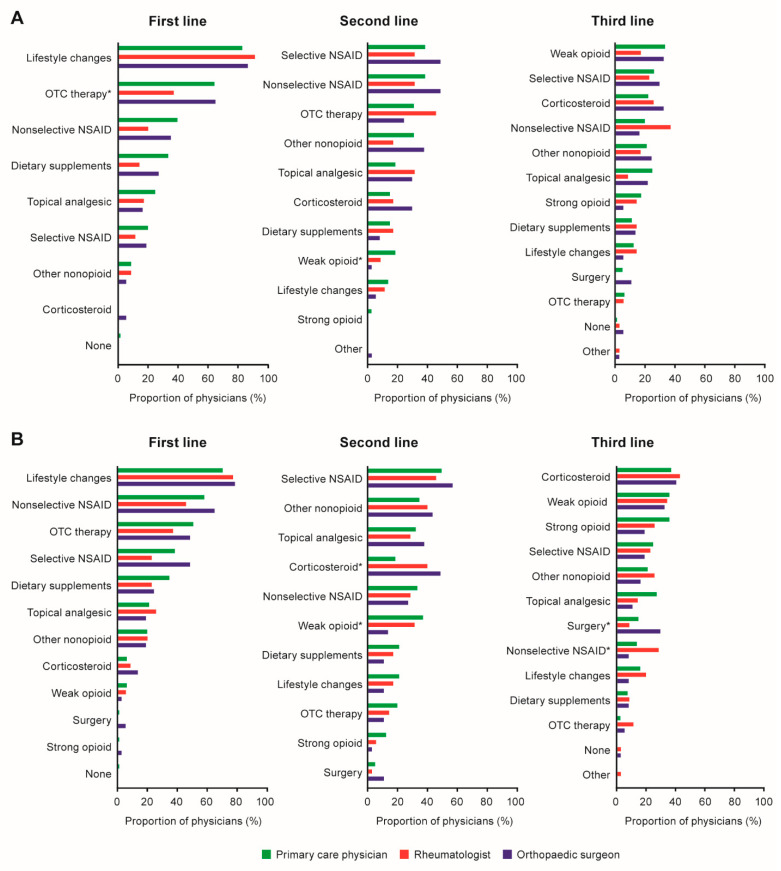
Treatment strategy for patients with (**A**) mild OA or (**B**) moderate/severe OA. * *p* < 0.05 for comparison across physician specialties. For data and fourth-line treatments, see Table S1 (mild OA) and Table S2 (moderate/severe OA). NSAID, nonsteroidal anti-inflammatory drug; OA, osteoarthritis; OTC, over the counter.

Physicians reported rates of recommended and/or prescribed opioid (weak or strong) use stratifying by line of therapy (first-, second-, or third-line) and across patients with mild and moderate/severe OA (Figure 2A, Table S1). Areas where rates differed by specialty included second-line use of weak opioids for patients with mild OA (18.5%, 8.6% and 2.7%; *p* = 0.0392) and moderate/severe OA (37.0%, 31.4% and 13.5%; *p* = 0.0347), for primary care physicians, rheumatologists, and orthopaedic surgeons, respectively. No one reported recommending and/or prescribing weak or strong opioids as first-line treatment for patients with mild OA.

### 3.4. Attributes of OA Medications

**Figure 3:**
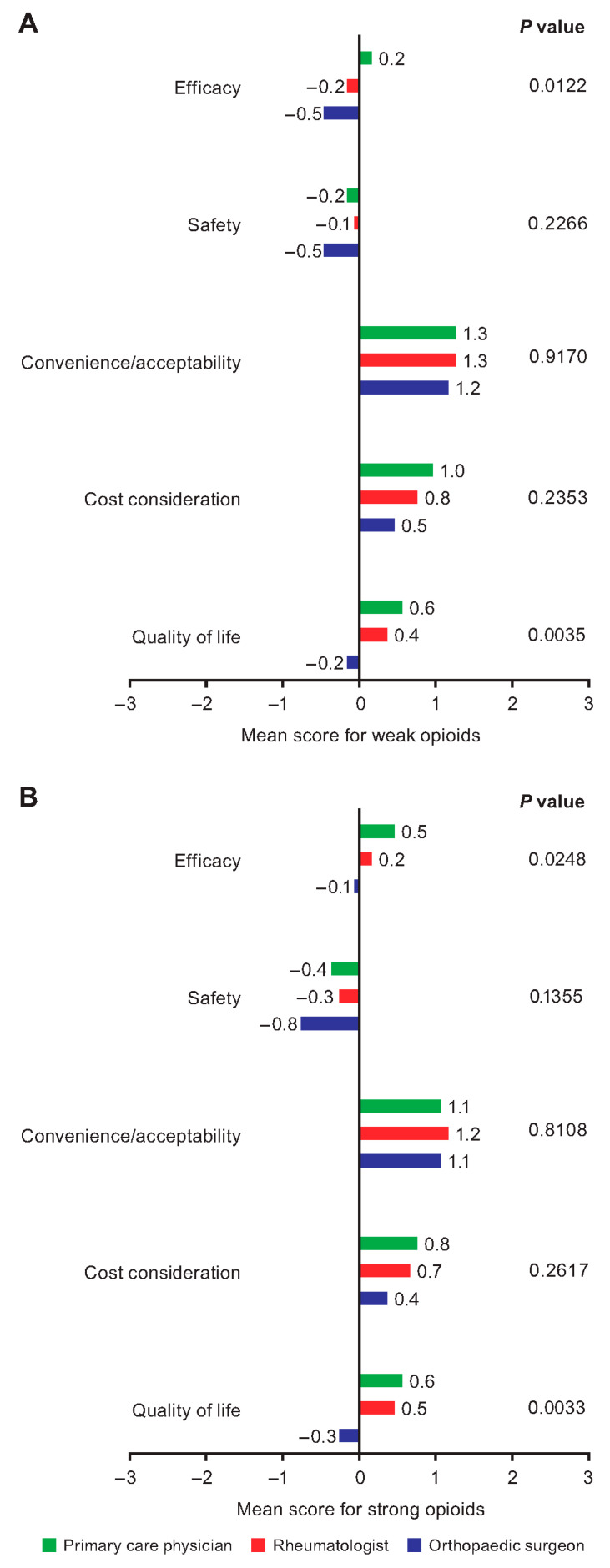
Physician ratings of the performance of (**A**) weak opioids and (**B**) strong opioids against attributes for the treatment of OA-related pain. *p* value for comparison across physician specialties. Attribute scores ranged from −3 (performs very badly) to +3 (performs very well). For attribute details and data for all medications, see Table S3. OA, osteoarthritis.

Medication attributes that differed by specialty included ratings given for efficacy and quality of life for both weak and strong opioids (Figure 3). There were no significant differences across specialties in the ratings given for safety, convenience/acceptability, and cost of either weak opioids or strong opioids (Figure 3).

Considering other medications for OA, ratings for the efficacy, safety, convenience/acceptability, cost, and quality of life associated with NSAIDs and topical analgesics were not significantly different across specialties (Table S3). Ratings for the efficacy of nonopioid analgesics (*p* = 0.0410) and cost considerations for injectable corticosteroids (*p* = 0.0482) differed significantly across specialties (Table S3).

### 3.5. Barriers to Opioid Prescribing

**Figure 4:**
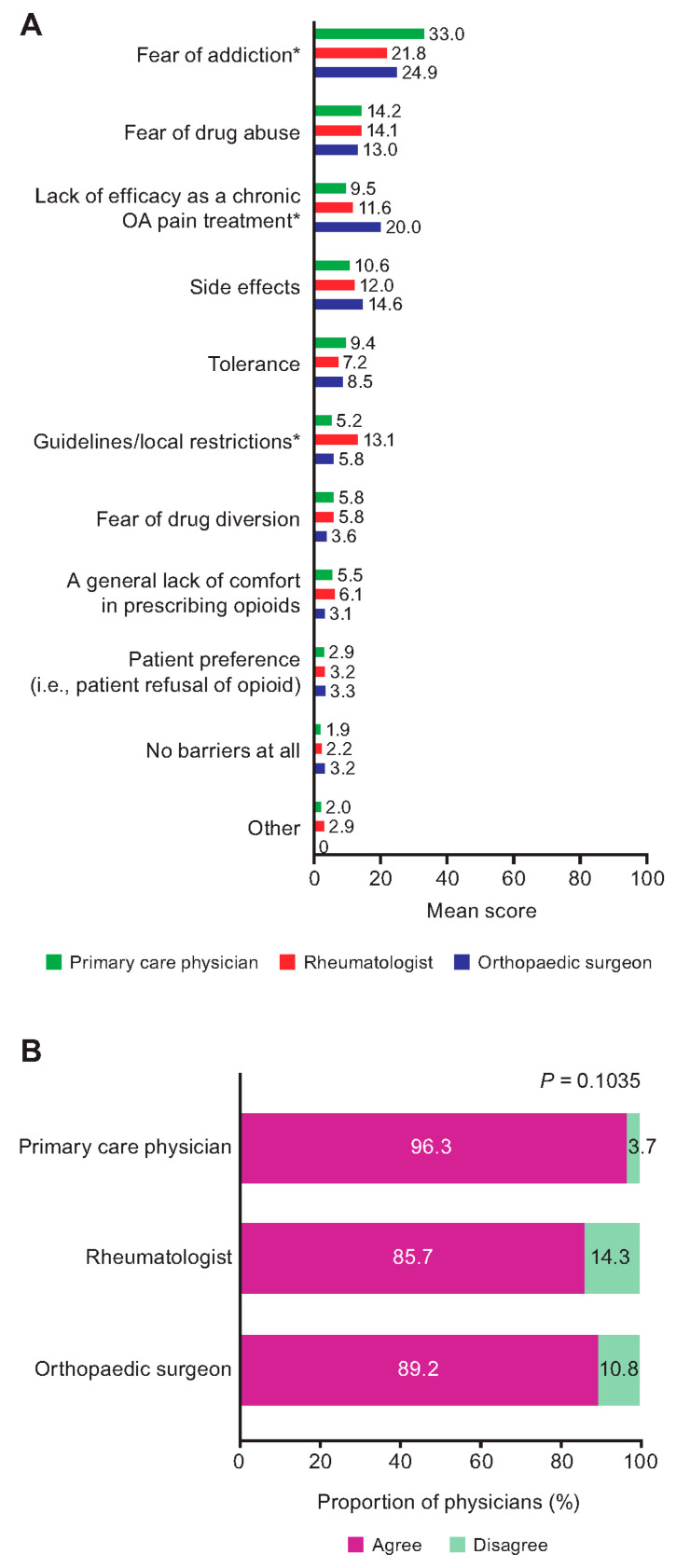
Physician perceptions related to the use of opioids: (**A**) barriers to prescribing and (**B**) concerns about dependence. * *p* < 0.05. *p* value for comparison across physician specialties. For data, see Table S4. OA, osteoarthritis.

Across specialties, physicians generally agreed on the barriers to opioid prescribing, with fear of addiction and fear of drug abuse being the most important (Figure 4A). Significant differences across the specialties included their consideration of fear of addiction (mean score 33.0, 21.8 and 24.9 for primary care physicians, rheumatologists and orthopaedic surgeons, respectively [*p* = 0.0459]), lack of efficacy as a chronic OA pain treatment (mean score 9.5, 11.6 and 20.0, respectively [*p* = 0.0157]) and guidelines/local restrictions (mean score 5.2, 13.1 and 5.8, respectively [*p* = 0.0212]) as barriers to opioid prescribing (Figure 4A, Table S4; the maximum score for most important = 100).

Overall, 92.2% of physicians were concerned about drug dependence, with no significant difference across specialties (Figure 4B). Overall, 56.9% of physicians had never heard of a dose-sparing approach to opioid prescribing, with no significant difference across specialties (Table 2).

## 4. Discussion

In this study, primary care physicians, rheumatologists and orthopaedic surgeons reported changes in their opioid prescribing patterns for OA one year following the publication of the CDC 2016 guideline [19,20]. Almost half of the physicians who reported prescribing opioids in the past year said they had decreased opioid prescribing, and the majority of those who reported changing their opioid prescribing attributed the changes to the CDC 2016 guideline.

Among those physicians who reported they had prescribed opioids in the previous year, approximately half reported no change in their opioid prescribing; the reason(s) for this are not clear, but it is possible that these physicians were not aware of the CDC 2016 guideline change. There were no significant differences across specialties in reported changes in opioid prescribing behaviour nor the impact of the CDC 2016 guideline [19,20]. Of the primary care physicians who reported changes in their opioid prescribing behaviour, approximately 1 in 6 were unaware of the CDC 2016 guideline change [19,20]. Although the guideline focused on primary care [19,20], orthopaedic surgeons reported considerable compliance with it. Small proportions of physicians across all three specialties reported increased opioid prescribing, although it is not clear if they were unaware of the guideline or if other factors (such as patient characteristics) may have influenced this behaviour. The CDC provided clinical tools, including a mobile application and training, to facilitate appropriate implementation [24].

Traditionally, strong opioids were reserved for severe pain that was not responsive to other analgesics, although greater understanding of pain physiology has since resulted in a less linear, more flexible approach to treatment [25]. In the current study, both weak and strong opioids were most likely to be prescribed for patients with severe OA, and only small proportion of patients with mild OA were prescribed any opioid. There were few differences across specialties in this respect, except for prescribing of weak opioids for patients with severe OA.

Physicians across the three specialties generally agreed on treatment strategies for OA, and reported that nonopioid options were prioritized, in line with treatment guidelines [8,9,10,11]. They reported that opioids were infrequently prescribed first line, and strong opioids were mostly considered a third-line treatment for OA. A database study found 17.6% of patients with knee OA were initiated on opioids, [26] which is a higher proportion of patients than is reflected in the first-line treatment strategies in the current study. Direct comparison is not possible, however, because the current data were based on physician report whereas the database study was based on electronic health records, [26] and there were also differences in patient population (including affected joints) and timeframes. There were significant differences across specialties in the current study with respect to the second-line use of weak opioids, for both mild OA and moderate/severe OA (primary care physicians reported greater use than rheumatologists, and the lowest use was reported by orthopaedic surgeons). The reason for this is unclear but could be influenced by differences in patient populations across the specialties, since opioid use has been associated with comorbidities (including gastrointestinal disease) that might contraindicate alternative treatments (such as NSAIDs) [27]. Other factors have been reported to be associated with opioid use in patients with OA, including depressive symptoms and greater pain/disease burden [27,28].

Although the minimal efficacy of opioids for chronic OA pain is well established [3,4,5,6], perceptions of the efficacy of opioids differed across specialties in the current study, with primary care physicians viewing efficacy more favourably than rheumatologists, and orthopaedic surgeons having the worst view. These different perceptions of the efficacy of opioids could be influenced by differences in focus (in terms of OA pathogenesis or how they judge treatment success) within the treatment pathway, differences in knowledge of the evidence base for opioid efficacy or differences in patient populations (in terms of their underlying OA and/or response to treatments). A previous study also found that orthopaedists had low confidence in the efficacy of opioids for chronic pain [29]. In the current study, orthopaedic surgeons also had the worst view of the quality of life associated with opioids, and it is also noteworthy that approximately 1 in 4 reported they had not prescribed opioids for OA.

In the current study, most physicians were concerned about drug dependence, and fear of addiction and fear of drug abuse were the most important barriers to opioid prescribing identified across specialties. Similar concerns were previously reported for primary care physicians with respect to prescribing opioids for chronic pain [30]. The risks of opioid-related harms are dose-dependent [19]. In the long term, a reduction in the number of patients prescribed high doses can be achieved by starting fewer patients on opioids and not escalating to high dosages [24]. For those already prescribed opioids, any dose tapering needs to be carefully considered and conducted slowly to minimise withdrawal symptoms [24]. However, patients with chronic pain can expect improvements in pain, function and quality of life following the reduction or discontinuation of opioids [31]. The CDC 2016 guideline advocates maximising the use of nonpharmacologic and nonopioid pharmacologic treatments, [19,20] but over half of all physicians in the current survey were unaware of opioid-sparing approaches (combining non-opioid analgesics and opioids and so that lower doses are needed). 

This study has some limitations. Information on prescribing practices was based on physician recall and report (rather than verifiable prescription numbers) and may be subject to social-desirability bias (over-reporting of “good” behaviour). The majority of the questionnaire in the current study related to the severity of OA rather than the severity of OA pain: although pain is a key consideration when physicians assess the overall severity of OA, multiple patient characteristics may be taken into account and the factors may differ across specialties [32]. The questionnaire relied on each physician’s definition of OA severity (mild vs. moderate vs. severe) and type of opioid (weak vs. strong). The analyses were largely descriptive (intended to generate hypotheses), and the Adelphi DSP methodology was designed to support clinical understanding of how diseases are managed in real-world settings and were not powered to address specific hypotheses [21]. Sample sizes may be too small to detect small to modest differences. There were minimal exclusion criteria for the selection of physicians, but inclusion could be influenced by willingness to participate. Most physicians were in private practice, so the data may not be representative of other practice settings. Not all physician specialties treating patients with OA were represented, and extrapolation to other specialties is not possible.

## 5. Conclusions

This study found that, among primary care physicians, rheumatologists and orthopaedic surgeons who reported prescribing opioids in the past year, almost half reported decreased opioid prescribing. The majority of those reporting a change in their opioid prescribing attributed the change to the CDC 2016 guideline. Physicians reported that both weak and strong opioids were most likely to be prescribed for patients with severe OA, and strong opioids were mostly considered third-line treatment. Perceptions of the efficacy and quality of life associated with opioids were significantly different across specialties. Physicians generally agreed on the barriers to opioid prescribing, with fear of addiction and fear of drug abuse being the most important. Updated analyses assessing recent opioid prescribing behaviours and associated patient treatment outcomes related to guideline changes are warranted, along with a detailed study of the reasons patients with OA are prescribed opioids.

## Figures and Tables

**Table 1:** Physician characteristics.

	Total (n = 153)	Primary Care Physician (n = 81)	Rheumatologist (n = 35)	Orthopaedic Surgeon (n = 37)
Male, n (%)	111 (72.5)	48 (59.3)	26 (74.3)	37 (100.0)
Years since specialty qualification, n (%)				
<4	9 (5.9)	2 (2.5)	0 (0.0)	7 (18.9)
4–14	25 (16.3)	11 (13.6)	8 (22.9)	6 (16.2)
15–24	46 (30.1)	25 (30.9)	8 (22.9)	13 (35.1)
≥25	73 (47.7)	43 (53.1)	19 (54.3)	11 (29.7)
Proportion of patients seen in setting, mean % (SD)				
Private	84.8 (29.3)	83.8 (32.5)	94.9 (10.9)	77.2 (31.7)
Public	14.1 (28.4)	14.5 (31.1)	4.8 (10.3)	22.1 (31.9)
Hospital	11.6 (20.0)	7.4 (16.2)	5.5 (8.1)	26.6 (27.3)
Office	87.3 (21.3)	90.9 (19.4)	94.3 (8.4)	72.7 (26.9)
Other	1.1 (8.4)	1.7 (11.2)	0.3 (1.2)	0.7 (4.1)
Proportion of patients by physician-perceived OA severity, mean % (SD)				
Mild	34.6 (16.5)	39.3 (17.4)	24.7 (12.5)	33.9 (13.9)
Moderate	41.6 (12.7)	39.6 (13.1)	47.3 (11.5)	40.6 (11.7)
Severe	23.7 (11.5)	21.1 (11.4)	28.0 (10.9)	25.5 (10.8)
Always follow OA treatment guidelines, n (%)	70 (45.8)	39 (48.1)	11 (31.4)	20 (54.1)

OA, osteoarthritis; SD, standard deviation.

**Table 2:** Physician prescribing of opioids for OA.

	Total (n = 153)	Primary Care Physician (n = 81)	Rheumatologist (n = 35)	Orthopaedic Surgeon (n = 37)
Prescribed opioids for OA in the past year, n (% yes) ***	139 (90.8)	78 (96.3)	34 (97.1)	27 (73.0)
Proportion of patients prescribed weak opioid, mean % (SD)				
Mild OA	5.3 (11.4)	6.1 (12.7)	3.9 (6.4)	5.0 (12.3)
Moderate OA	15.6 (14.2)	16.4 (13.7)	16.9 (13.7)	12.5 (15.5)
Severe OA **	25.3 (19.5)	28.2 (19.2)	28.4 (22.2)	15.9 (14.5)
Proportion of patients prescribed strong opioid, mean % (SD)				
Mild OA	2.3 (7.8)	2.0 (5.2)	1.9 (4.9)	3.3 (13.2)
Moderate OA	9.9 (12.4)	11 (13.9)	10.6 (11.1)	6.8 (9.8)
Severe OA	23.7 (22.0)	26.2 (21.1)	25.7 (23.1)	16.6 (21.8)
Opioid dose-sparing approach ever adopted, n (%)				
Yes	34 (22.2)	16 (19.8)	10 (28.6)	8 (21.6)
No	32 (20.9)	13 (16.0)	10 (28.6)	9 (24.3)
never heard of this approach	87 (56.9)	52 (64.2)	15 (42.9)	20 (54.1)

*** *p* value < 0.001; ** *p* < 0.01; for comparison across physician specialties. OA, osteoarthritis; SD, standard deviation.

## Data Availability

The data that support the findings of this study are available from Adelphi Real World, but restrictions apply to the availability of these data, which were used under license for the current study and so are not publicly available. However, data are available from the authors upon reasonable request and with permission from Adelphi Real World.

## Supplementary Materials

The following supporting information can be downloaded at: https://www.mdpi.com/article/10.3390/jcm12020589/s1, Table S1. Treatment strategy for patients with mild OA; Table S2. Treatment strategy for patients with moderate/severe OA; Table S3. Physician ratings of medication performance against attributes for the treatment of OA-related pain; Table S4. Physician perceptions related to the use of opioids: barriers to prescribing.
