# Supplementary material for: Opioid Prescribing for Osteoarthritis: Cross-Sectional Survey among Primary Care Physicians, Rheumatologists, and Orthopaedic Surgeons
Source: J Clin Med. 2023 Jan 11;12(2):589. doi: 10.3390/jcm12020589 (PMC9864807; doi:10.3390/jcm12020589)
Supplement: Supplementary file 1 [file jcm-12-00589-s001.zip › jcm-2080709-supplementary.pdf]

Journal of
Clinical Medicine

MDPI

Table S1. Treatment strategy for patients with mild OA

	Primary care physician (n = 81)	Rheumatologist (n = 35)	Orthopaedic surgeon (n = 37)	*P* value
**First-line treatment for mild OA**				
Lifestyle changes, e.g., diet/exercise	67 (82.7)	32 (91.4)	32 (86.5)	.4638
Over-the-counter therapy	52 (64.2)	13 (37.1)	24 (64.9)	.0162
Dietary supplements	27 (33.3)	5 (14.3)	10 (27.0)	.1077
Nonselective NSAID	32 (39.5)	7 (20.0)	13 (35.1)	.1241
Selective NSAID	16 (19.8)	4 (11.4)	7 (18.9)	.5434
Other nonopioid analgesic	7 (8.6)	3 (8.6)	2 (5.4)	.8524
Corticosteroid	0 (0.0)	0 (0.0)	2 (5.4)	.1084
Topical analgesic	20 (24.7)	6 (17.1)	6 (16.2)	.4739
None	1 (1.2)	0 (0.0)	0 (0.0)	1
**Second-line treatment for mild OA**				
Lifestyle changes, e.g., diet/exercise	11 (13.6)	4 (11.4)	2 (5.4)	.4623
Over-the-counter therapy	25 (30.9)	16 (45.7)	9 (24.3)	.1354
Dietary supplements	12 (14.8)	6 (17.1)	3 (8.1)	.5228
Nonselective NSAID	31 (38.3)	11 (31.4)	18 (48.6)	.3163
Selective NSAID	31 (38.3)	11 (31.4)	18 (48.6)	.3163
Other nonopioid analgesic	25 (30.9)	6 (17.1)	14 (37.8)	.1433
Weak opioid analgesic	15 (18.5)	3 (8.6)	1 (2.7)	.0392
Strong-acting opioid analgesic	2 (2.5)	0 (0.0)	0 (0.0)	1
Corticosteroid	12 (14.8)	6 (17.1)	11 (29.7)	.1515
Topical analgesic	15 (18.5)	11 (31.4)	11 (29.7)	.2187
Other (specify)	0 (0.0)	0 (0.0)	1 (2.7)	.4706
**Third-line treatment for mild OA**				
Lifestyle changes, e.g., diet/exercise	10 (12.3)	5 (14.3)	2 (5.4)	.4349
Over-the-counter therapy	5 (6.2)	2 (5.7)	0 (0.0)	.3999
Dietary supplements	9 (11.1)	5 (14.3)	5 (13.5)	.8489
Nonselective NSAID	16 (19.8)	13 (37.1)	6 (16.2)	.0667
Selective NSAID	21 (25.9)	8 (22.9)	11 (29.7)	.8008
Other nonopioid analgesic	17 (21.0)	6 (17.1)	9 (24.3)	.7553
Weak opioid analgesic	27 (33.3)	6 (17.1)	12 (32.4)	.192
Strong-acting opioid analgesic	14 (17.3)	5 (14.3)	2 (5.4)	.1971
Corticosteroid	18 (22.2)	9 (25.7)	12 (32.4)	.4978
Topical analgesic	20 (24.7)	3 (8.6)	8 (21.6)	.1362
Surgery	4 (4.9)	0 (0.0)	4 (10.8)	.0851
None	1 (1.2)	1 (2.9)	2 (5.4)	.3428
Other (specify)	0 (0.0)	1 (2.9)	1 (2.7)	.2198
**Fourth-line treatment for mild OA**				
Lifestyle changes, e.g., diet/exercise	7 (8.6)	2 (5.7)	0 (0.0)	.175

Over-the-counter therapy	3 (3.7)	2 (5.7)	0 (0.0)	.3365
Dietary supplements	4 (4.9)	3 (8.6)	3 (8.1)	.6884
Nonselective NSAID	3 (3.7)	4 (11.4)	1 (2.7)	.2179
Selective NSAID	15 (18.5)	8 (22.9)	2 (5.4)	.1
Other nonopioid analgesic	7 (8.6)	4 (11.4)	2 (5.4)	.6345
Weak opioid analgesic	16 (19.8)	4 (11.4)	7 (18.9)	.5434
Strong-acting opioid analgesic	30 (37.0)	8 (22.9)	13 (35.1)	.3194
Corticosteroid	29 (35.8)	8 (22.9)	6 (16.2)	.0659
Topical analgesic	15 (18.5)	3 (8.6)	4 (10.8)	.291
Surgery	36 (44.4)	13 (37.1)	20 (54.1)	.3487
None	4 (4.9)	2 (5.7)	5 (13.5)	.2752

Data are n (%) of physicians. *P* value for comparison across physician specialties.

NSAID, nonsteroidal anti-inflammatory drug; OA, osteoarthritis.

Table S2. Treatment strategy for patients with moderate/severe OA

	Primary care physician (n = 81)	Rheumatologist (n = 35)	Orthopaedic surgeon (n = 37)	*P* value
**First-line treatment for moderate/severe OA**				
Lifestyle changes, e.g., diet/exercise	57 (70.4)	27 (77.1)	29 (78.4)	.5777
Over-the-counter therapy	41 (50.6)	13 (37.1)	18 (48.6)	.4004
Dietary supplements	28 (34.6)	8 (22.9)	9 (24.3)	.3291
Nonselective NSAID	47 (58.0)	16 (45.7)	24 (64.9)	.2486
Selective NSAID	31 (38.3)	8 (22.9)	18 (48.6)	.0745
Other nonopioid analgesic	16 (19.8)	7 (20.0)	7 (18.9)	.9922
Weak opioid analgesic	5 (6.2)	2 (5.7)	1 (2.7)	.7968
Strong-acting opioid analgesic	1 (1.2)	0 (0.0)	1 (2.7)	.7214
Corticosteroid	5 (6.2)	3 (8.6)	5 (13.5)	.4374
Topical analgesic	17 (21.0)	9 (25.7)	7 (18.9)	.769
Surgery	1 (1.2)	0 (0.0)	2 (5.4)	.2764
None	1 (1.2)	0 (0.0)	0 (0.0)	1
**Second-line treatment for moderate/severe OA**				
Lifestyle changes, e.g., diet/exercise	17 (21.0)	6 (17.1)	4 (10.8)	.4029
Over-the-counter therapy	16 (19.8)	5 (14.3)	4 (10.8)	.4436
Dietary supplements	17 (21.0)	6 (17.1)	4 (10.8)	.4029
Nonselective NSAID	27 (33.3)	10 (28.6)	10 (27.0)	.7509
Selective NSAID	40 (49.4)	16 (45.7)	21 (56.8)	.6254
Other nonopioid analgesic	28 (34.6)	14 (40.0)	16 (43.2)	.6388
Weak opioid analgesic	30 (37.0)	11 (31.4)	5 (13.5)	.0347
Strong-acting opioid analgesic	10 (12.3)	2 (5.7)	1 (2.7)	.2106
Corticosteroid	15 (18.5)	14 (40.0)	18 (48.6)	.0018
Topical analgesic	26 (32.1)	10 (28.6)	14 (37.8)	.6948
Surgery	4 (4.9)	1 (2.9)	4 (10.8)	.3794
**Third-line treatment for moderate/severe OA**				
Lifestyle changes, e.g., diet/exercise	13 (16.0)	7 (20.0)	3 (8.1)	.3446
Over-the-counter therapy	2 (2.5)	4 (11.4)	2 (5.4)	.1034
Dietary supplements	6 (7.4)	3 (8.6)	3 (8.1)	1
Nonselective NSAID	11 (13.6)	10 (28.6)	3 (8.1)	.0435
Selective NSAID	20 (24.7)	8 (22.9)	7 (18.9)	.7867
Other nonopioid analgesic	17 (21.0)	9 (25.7)	6 (16.2)	.6122
Weak opioid analgesic	29 (35.8)	12 (34.3)	12 (32.4)	.9371
Strong-acting opioid analgesic	29 (35.8)	9 (25.7)	7 (18.9)	.1506
Corticosteroid	30 (37.0)	15 (42.9)	15 (40.5)	.8256
Topical analgesic	22 (27.2)	5 (14.3)	4 (10.8)	.0741
Surgery	12 (14.8)	3 (8.6)	11 (29.7)	.0431
None	0 (0.0)	1 (2.9)	1 (2.7)	.2198

Other (specify)	0 (0.0)	1 (2.9)	0 (0.0)	.2288
**Fourth-line treatment for moderate/severe OA**				
Lifestyle changes, e.g., diet/exercise	12 (14.8)	5 (14.3)	1 (2.7)	.1218
Over-the-counter therapy	3 (3.7)	2 (5.7)	0 (0.0)	.3365
Dietary supplements	4 (4.9)	3 (8.6)	2 (5.4)	.7449
Nonselective NSAID	4 (4.9)	3 (8.6)	0 (0.0)	.1618
Selective NSAID	6 (7.4)	8 (22.9)	1 (2.7)	.0125
Other nonopioid analgesic	6 (7.4)	5 (14.3)	0 (0.0)	.0511
Weak opioid analgesic	12 (14.8)	7 (20.0)	6 (16.2)	.7861
Strong-acting opioid analgesic	30 (37.0)	18 (51.4)	16 (43.2)	.3464
Corticosteroid	32 (39.5)	7 (20.0)	4 (10.8)	.0027
Topical analgesic	10 (12.3)	5 (14.3)	3 (8.1)	.7117
Surgery	49 (60.5)	23 (65.7)	24 (64.9)	.8275
None	3 (3.7)	0 (0.0)	4 (10.8)	.0891

Data are n (%) of physicians. *P* value for comparison across physician specialties

NSAID, nonsteroidal anti-inflammatory drug; OA, osteoarthritis.

Table S3. Physician ratings of medication performance against attributes for the treatment of OA-related pain

Attribute	Medication	Primary care physician (n = 81)	Rheumatologist (n = 35)	Orthopaedic surgeon (n = 37)	*P* value
Efficacy†	Overall medications	0.3 (0.74)	–0.0 (0.80)	–0.1 (0.92)	.0195
	NSAID	0.3 (0.95)	0.0 (1.00)	0.0 (0.90)	.1009
	Nonopioid analgesic	0.1 (1.02)	–0.2 (0.92)	–0.4 (1.01)	.0410
	Weak opioid	0.2 (0.95)	–0.2 (1.10)	–0.5 (1.32)	.0122
	Strong opioid	0.5 (1.03)	0.2 (1.23)	–0.1 (1.40)	.0248
	Injectable corticosteroid	1.1 (1.04)	0.8 (0.97)	0.9 (0.94)	.3579
	Topical analgesic	–0.3 (1.19)	–0.6 (1.14)	–0.6 (1.38)	.2545
Safety‡	Overall medications	0.3 (0.79)	0.3 (0.99)	0.3 (0.88)	.9588
	NSAID	0.1 (1.03)	–0.1 (1.15)	0.2 (1.14)	.4220
	Nonopioid analgesic	0.4 (1.09)	0.4 (1.22)	0.5 (1.22)	.9768
	Weak opioid	–0.2 (1.17)	–0.1 (1.23)	–0.5 (1.19)	.2266
	Strong opioid	–0.4 (1.09)	–0.3 (1.27)	–0.8 (1.18)	.1355
	Injectable corticosteroid	0.7 (1.03)	0.8 (1.26)	0.9 (0.97)	.4597
	Topical analgesic	1.6 (1.00)	1.5 (1.17)	1.7 (1.13)	.8286
Convenience/ acceptability§	Overall medications	1.4 (0.82)	1.3 (0.91)	1.4 (0.96)	.9591
	NSAID	1.8 (0.94)	1.5 (0.98)	1.7 (1.03)	.3250
	Nonopioid analgesic	1.6 (0.99)	1.5 (1.04)	1.6 (1.12)	.8941
	Weak opioid	1.3 (1.06)	1.3 (1.13)	1.2 (1.21)	.9170
	Strong opioid	1.1 (1.07)	1.2 (1.03)	1.1 (1.37)	.8108
	Injectable corticosteroid	0.9 (0.99)	1.1 (1.02)	1.0 (0.90)	.6614
	Topical analgesic	1.2 (1.10)	1.2 (0.97)	1.3 (0.96)	.9395
Cost consideration¶	Overall medications	1.0 (0.93)	0.8 (1.14)	0.9 (1.00)	.4996
	NSAID	1.5 (1.13)	1.0 (1.16)	1.4 (1.04)	.1018
	Nonopioid analgesic	1.4 (1.17)	0.9 (1.39)	1.5 (1.12)	.0624
	Weak opioid	1.0 (1.31)	0.8 (1.24)	0.5 (1.48)	.2353
	Strong opioid	0.8 (1.35)	0.7 (1.32)	0.4 (1.62)	.2617
	Injectable corticosteroid	0.4 (1.28)	1.1 (1.35)	0.7 (1.62)	.0482
	Topical analgesic	0.6 (1.53)	0.2 (1.60)	0.7 (1.60)	.3328
Quality of life††	Overall medications	0.8 (0.86)	0.5 (0.93)	0.4 (1.13)	.0884
	NSAID	0.9 (1.02)	0.4 (1.22)	0.5 (1.19)	.1045
	Nonopioid analgesic	0.7 (1.10)	0.5 (0.98)	0.4 (1.26)	.3677
	Weak opioid	0.6 (1.11)	0.4 (1.06)	–0.2 (1.36)	.0035
	Strong opioid	0.6 (1.16)	0.5 (1.09)	–0.3 (1.54)	.0033
	Injectable corticosteroid	1.3 (1.00)	1.1 (0.99)	1.2 (1.18)	.7907
	Topical analgesic	0.6 (1.20)	0.3 (1.30)	0.6 (1.57)	.5642

Data are mean score (standard deviation) of attributes within each category with scores ranging per attribute from –3 (performs very badly) to +3 (performs very well). *P* value for comparison across physician specialties.

†Mean of 8 attributes: effective relief of pain at rest, effective relief of pain on movement, reduces number of tender/swollen joints, maintains symptomatic relief over time without need to increase dose/alter frequency, rapid onset of action, effective in controlling acute flares, slows disease progression and, delays need for surgery.
‡Mean of 9 attributes: safe long-term use, good gastrointestinal safety, low incidence of serious adverse events/severe side effects, suitable for patients with cardiovascular risk, can be used continuously (without a drug break), appropriate for use in the elderly (>65 years), no need for frequent monitoring/testing, low risk of addiction/abuse and, can be used in combination with other pain drugs.
§Mean of 6 attributes: ease of administration for patient, convenient dosing schedule, physician familiarity/experience, on formulary/hospital approved drug list, can be used in combination with other OA therapies and, accordance with treatment guidelines.
¶Mean of 2 attributes: low out of pocket cost/affordability for patient and, low cost/affordable for physician/practice.
††Mean of 6 attributes: improves/maintains attendance at work, improves/maintains ability to perform activities of daily living/leisure activities, improves/maintains relationships/social life, improves patient functionality, improves patient's sleep patterns/quality of sleep and, improves/maintains patient's independence.
NSAID, nonsteroidal anti-inflammatory drug; OA, osteoarthritis.

Table S4. Physician perceptions related to the use of opioids: barriers to prescribing

Characteristics	Primary care physician (n = 81)	Rheumatologist (n = 35)	Orthopaedic surgeon (n = 37)	*P* value
Barriers to opioid prescribing				
Fear of addiction	33.0 (24.27)	21.8 (24.86)	24.9 (23.67)	.0459
Fear of drug abuse	14.2 (15.51)	14.1 (12.48)	13.0 (10.17)	.8996
Lack of efficacy as a chronic OA pain treatment	9.5 (13.94)	11.6 (16.03)	20.0 (26.67)	.0157
Side effects	10.6 (10.14)	12.0 (10.74)	14.6 (17.22)	.2696
Tolerance	9.4 (9.92)	7.2 (8.38)	8.5 (11.95)	.5574
Guidelines/local restrictions	5.2 (9.52)	13.1 (23.01)	5.8 (11.64)	.0212
Fear of drug diversion	5.8 (12.50)	5.8 (7.63)	3.6 (4.66)	.5209
General lack of comfort in prescribing opioids	5.5 (13.65)	6.1 (7.08)	3.1 (7.11)	.4438
Patient preference (i.e., patient refusal of opioid)	2.9 (4.72)	3.2 (4.75)	3.3 (4.27)	.8892
Other	2 (12.89)	2.9 (16.9)	–	.5947
No barriers at all	1.9 (6.57)	2.2 (7.71)	3.2 (11.01)	.6873

Data are mean score (standard deviation) of physician allocated points out of 100 distributed based on level of importance across the list of attributes such that higher scores =greater perceived barrier. *P* value for comparison across physician specialties.
OA, osteoarthritis.
